# Advanced modeling of pharmaceutical solubility in solvents using artificial intelligence techniques: assessment of drug candidate for nanonization processing

**DOI:** 10.3389/fmed.2024.1435675

**Published:** 2024-07-22

**Authors:** Turki Al Hagbani, Sameer Alshehri, Sami Bawazeer

**Affiliations:** ^1^Department of Pharmaceutics, College of Pharmacy, University of Hail, Hail, Saudi Arabia; ^2^Department of Pharmaceutics and Industrial Pharmacy, College of Pharmacy, Taif University, Taif, Saudi Arabia; ^3^Department of Pharmaceutical Sciences, Faculty of Pharmacy, Umm Al-Qura University, Makkah, Saudi Arabia

**Keywords:** drug development, solubility prediction, optimization, machine learning, modeling

## Abstract

This research is an analysis of multiple regression models developed for predicting ketoprofen solubility in supercritical carbon dioxide under different levels of T(K) and P(bar) as input features. Solubility of the drug was correlated to pressure and temperature as major operational variables. Selected models for this study are Piecewise Polynomial Regression (PPR), Kernel Ridge Regression (KRR), and Tweedie Regression (TDR). In order to improve the performance of the models, hyperparameter tuning is executed utilizing the Water Cycle Algorithm (WCA). Among, the PPR model obtained the best performance, with an R^2^ score of 0.97111, alongside an MSE of 1.6867E-09 and an MAE of 3.01040E-05. Following closely, the KRR model demonstrated a good performance with an R^2^ score of 0.95044, an MSE of 2.5499E-09, and an MAE of 3.49707E-05. In contrast, the TDR model produces a lower R^2^ score of 0.84413 together with an MSE of 7.4249E-09 and an MAE of 5.69159E-05.

## 1 Introduction

Simulation and modeling of pharmaceutical processes are great tools for development of pharmaceutical manufacturing and would help for shifting from batch toward continuous manufacture mode. Process analytical technology (PAT) and process modeling are important elements of process understanding for design of continuous manufacturing in pharmaceutical area (1–3) where they can be exploited to enhance the efficiency of process and products quality. Process modeling can be performed by finding the relationships between the process parameters and critical quality attributes of finished products. Once the relationship has been established, one can implement Quality-by-Design (QbD) for improvement of process and products (4, 5). Thus, development of robust and rigorous models for pharmaceutical processing is a major challenge which should be addressed.

There are different processing routes for manufacture of solid-dosage oral products which can be optimized via process modeling and theoretical computations. For instance, the method of wet granulation can be simulated via population balance model (PBM) to predict the granule size distribution. As the granules’ properties can affect the tablet characteristics, building relationship between granule size and tablet properties can be done via process modeling which would help for process understanding (6–8).

One of the main problems in pharmaceutical area is the poor solubility of APIs (Active Pharmaceutical Ingredients) in aqueous media which makes patients to take more dosage of drugs to obtain the therapeutics effectiveness. Taking more dosages would consequently result in side effects for patients. Therefore, some techniques have been developed to enhance the solubility of APIs in aqueous media such as production of nanomedicines (9–11). The processing of drugs with supercritical carbon dioxide has been reported as one of the methods for creation of nanomedicines. This method has attracted much attention and can be considered as a green method for preparation of nanosized APIs particles. Some computational models have been developed for description of this process, where the majority of studies focus on correlation of drug solubility dataset (12–15). Machine learning models are among the most commonly used methods for correlation of solubility data which can be used for a given dataset (16, 17).

Modern data analysis now heavily depends on machine learning (ML), which provides strong instruments and methods for deriving important insights from large datasets. ML includes a range of algorithms that let computers learn from data and, in the absence of explicit programming, make predictions or decisions. These algorithms can identify patterns, relationships, and trends within data, making ML particularly useful for tasks such as regression, classification, clustering, and anomaly detection (18, 19). The ML models have been used recently for correlation of pharmaceutical solubility in supercritical solvents such as CO_2_. Abouzied et al. (20) investigated the drug solubility in supercritical CO_2_ using multi-layer perceptron, k-nearest neighbors, and GPR methods. A great fitting accuracy was obtained with R^2^ more than 0.99 which confirmed the validity of ML models in estimating drug solubility. Support vector machine (SVM) has been one of the major methods used for evaluation of drug solubility in supercritical solvent which is useful in this area with great fitting accuracy (21).

In this paper, we focus on the application of ML to model the solubility of ketoprofen in supercritical carbon dioxide, a critical factor in pharmaceutical manufacturing processes. Accurate modeling of solubility is essential for optimizing production efficiency and ensuring the quality of pharmaceutical products. To achieve this, we employed three regression techniques:

1. Piecewise Polynomial Regression (PPR): PPR partitions the input data range into segments and applies polynomial functions to each segment, enabling a flexible and localized estimation of the regression function.
2. Kernel Ridge Regression (KRR): KRR combines ridge regression with kernel functions, enabling it to model non-linear relationships in the data by projecting it into higher-dimensional spaces.
3. Tweedie Regression (TDR): TDR is a generalized linear model capable of accommodating various distribution types, rendering it well-suited for modeling continuous, non-negative data with variance scaling with a power of the mean.

To optimize the performance of these regression models, we utilized the Water Cycle Algorithm (WCA), a nature-inspired optimization method that simulates the water cycle process to find optimal solutions. WCA has proven effective in navigating complex search spaces and identifying optimal parameter settings for various ML models.

The selected models are highly suitable for small datasets because of their adaptability and resilience in capturing complex relationships. PPR enables localized polynomial fits that can adapt to specific segments of the data, KRR effectively handles non-linearities even with limited data points using kernel functions, and TDR is designed for modeling continuous non-negative data with scalable variance. The careful application of these models, along with the Water Cycle Algorithm (WCA) for optimal parameter tuning, ensures that they are capable of delivering accurate and reliable solubility predictions despite the small dataset size.

Multiple contributions are made by this paper. First, we evaluate the three regression models for ketoprofen solubility prediction, highlighting their pros and cons. Second, we demonstrate how the Water Cycle Algorithm improves model performance by tuning hyperparameters. Finally, we present detailed dataset visualizations and statistical analyses to reveal T(K), P(bar), and solubility relationships. This study uses advanced ML techniques and robust optimization strategies to predict supercritical fluid solubility and advance pharmaceutical process optimization. Indeed, Piecewise Polynomial Regression (PPR), Kernel Ridge Regression (KRR), and Tweedie Regression (TDR) were used for the first time with the Water Cycle Algorithm (WCA) optimizer to improve the prediction accuracy of models for solubility of ketoprofen in supercritical CO_2_. The models are then used for evaluation of effect of temperature and pressure on the solubility variations.

## 2 Data set description

The dataset includes solubility measurements at temperatures spanning from 308.15 K to 338.15 K and pressures ranging from 160 bar to 400 bar, sourced from (22). The entire data points of the dataset are shown in Table 1. So, T and P are taken as inputs of ML models, and the drug solubility has been considered as the single output for all models in this study.

**Table 1:** Complete dataset of ketoprofen solubility in supercritical carbon dioxide (22).

T (K)	P (bar)	Solubility
308.15	160	2.21 × 10^−5^
	200	2.56 × 10^−5^
	240	2.87 × 10^−5^
	280	3.21 × 10^−5^
	320	3.45 × 10^−5^
	360	4.23 × 10^−5^
	400	4.56 × 10^−5^
318.15	160	5.01 × 10^−5^
	200	6.58 × 10^−5^
	240	7.68 × 10^−5^
	280	9.01 × 10^−5^
	320	1.12 × 10^−4^
	360	1.03 × 10^−4^
	400	1.20 × 10^−4^
328.15	160	7.01 × 10^−5^
	200	1.16 × 10^−4^
	240	1.75 × 10^−4^
	280	2.20 × 10^−4^
	320	2.54 × 10^−4^
	360	3.24 × 10^−4^
	400	3.59 × 10^−4^
338.15	160	1.01 × 10^−4^
	200	2.45 × 10^−4^
	240	3.25 × 10^−4^
	280	4.92 × 10^−4^
	320	5.79 × 10^−4^
	360	6.87 × 10^−4^
	400	7.12 × 10^−4^

**Figure 1:**
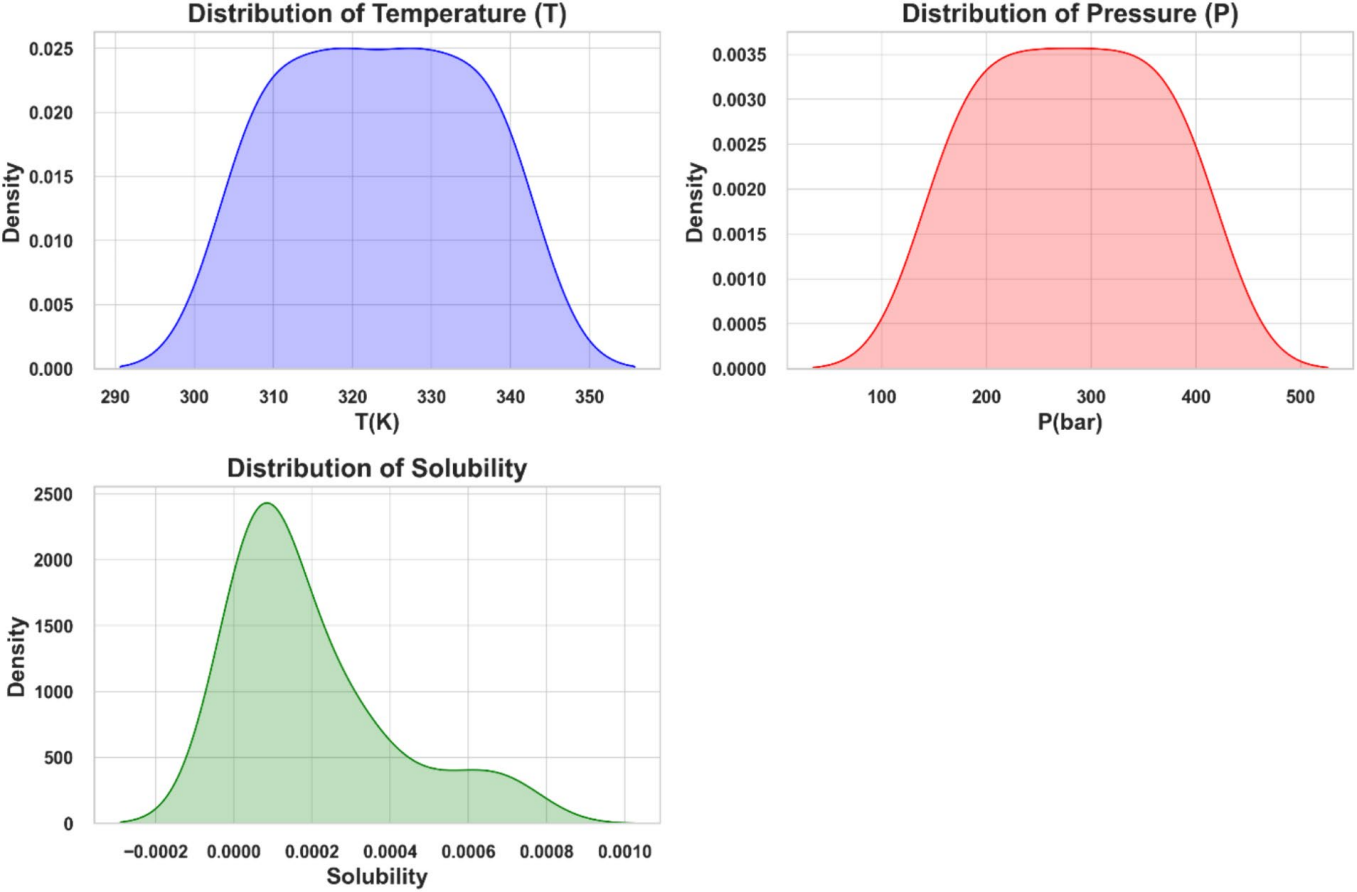
Distribution plots of temperature (T), pressure (P), and solubility of ketoprofen in supercritical CO_2_.

Figure 1 depicts distribution plots for temperature (T), pressure (P), and ketoprofen solubility in supercritical carbon dioxide. These plots use kernel density estimates (KDE) to represent the probability density functions of the variables. The solubility distribution is right-skewed, indicating that higher solubility values occur less frequently in the dataset.

**Figure 2:**
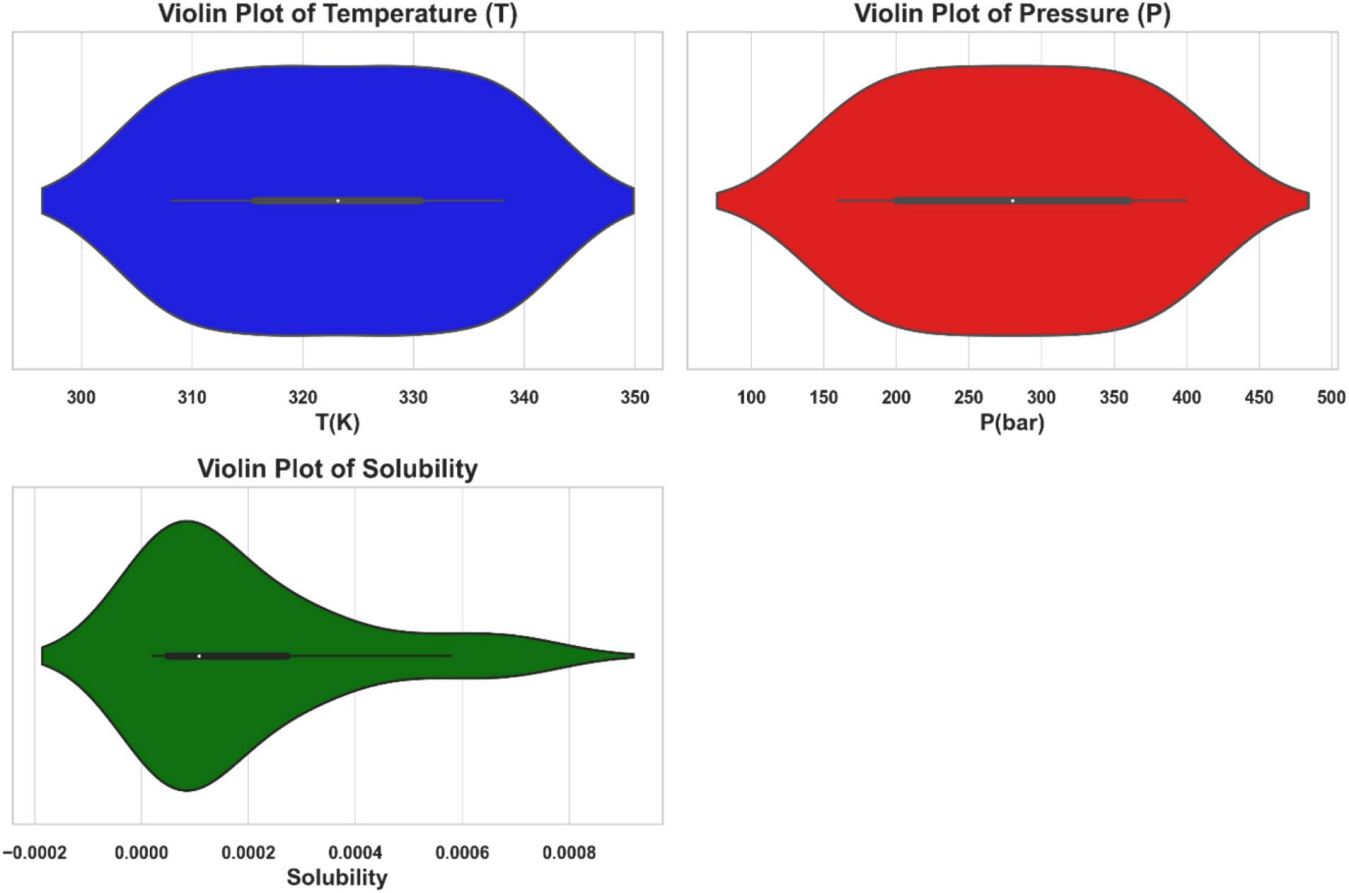
Violin plots illustrating the distributions of temperature (T), pressure (P), and the solubility of ketoprofen in supercritical CO_2_.

Furthermore, the violin plots in Figure 2 depict the temperature (T), pressure (P), and solubility of ketoprofen in supercritical carbon dioxide. Each plot combines a boxplot with a kernel density estimate (KDE). The KDE component shows the probability density of the data at various values, whereas the boxplot component within the violin plot displays the median, interquartile range, and potential outliers. The temperature and pressure data have relatively uniform distributions, whereas the solubility data has a more skewed distribution, indicating that solubility values vary across experimental conditions.

## 3 Methodology

### 3.1 Water cycle algorithm

Inspired by the natural water cycle, the Water Cycle Algorithm (WCA) is a population-based optimization algorithm. The method relies on the water cycle, which includes evaporation, cloud formation, and precipitation. The WCA replicates this cycle to find the best solutions. Initialization, evaporation, precipitation, and river formation are key algorithm steps (23, 24).

During the initialization phase, a set of potential solutions is generated in a random manner. In this context, every solution is defined by a set of hyper-parameter values. For instance, in a function optimization scenario, these parameters may represent the input variable values (25).

The evaluation of the solutions’ fitness values takes place in the evaporation phase. The fitness of a solution reflects its quality, with higher fitness values corresponding to superior solutions. These fitness values play a role in computing the evaporation rate, dictating the amount of water that evaporates from each solution (25, 26).

In the precipitation stage, the evaporated water is converted into clouds, which are subsequently dispersed randomly among the solution population. Each cloud symbolizes a potential enhancement to a solution. The fitness values of the clouds are assessed, and the most superior cloud is selected (27). The introduced steps of WCA are reiterated until a termination condition is met. The stopping criterion could involve attaining an specific fitness value, reaching a maximum number of iterations, or consuming a maximum computational time (24, 28).

**Figure 3:**
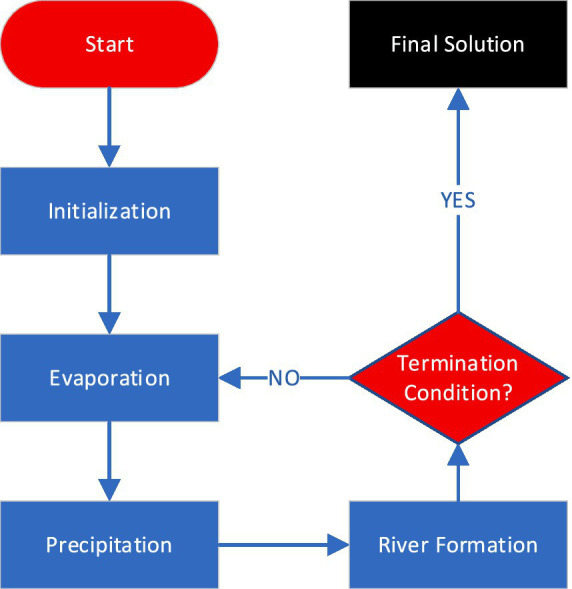
Water cycle algorithm (WCA).

Figure 3 shows the basic workflow of WCA algorithm. One of the strengths of the WCA is its capability to address multiple objectives. Multi-objective optimization finds the best solutions for conflicting goals (accuracy and generality in ML tasks). The dominance principle is used to extend the WCA to multiple goals. A solution dominates another solution if it excels in at least one objective without being inferior in any. Utilizing this idea, the WCA can identify a collection of solutions that are independent of one another (28). Natural-inspired processes give the Water Cycle Algorithm (WCA) benefits over Particle Swarm Optimization (PSO) and Differential Evolution (DE). WCA’s iterative evaporation, cloud formation, and river construction balance exploration and exploitation to avoid local optima and increase convergence. Its self-adjusting evaporation rates and cloud dispersal improve performance without parameter adjustment. The robust diversification mechanism of WCA provides constant exploration of new regions, while its structured navigation of complex search spaces via river formation efficiently avoids suboptimal regions. These properties make WCA more adaptable and effective than PSO and DE.

This algorithm is used for model optimization (hyper-parameter tuning) in this study. We used deterministic optimization here while there exists methods for optimization under uncertainty (29, 30). Here, each solution consists of a combination of hyper-parameter values and one of the objective functions is the RMSE error rate of the model build on each solution which should be maximized.

Also, by selecting the architecture with the least Akaike Information Criterion (AIC) value, the models are filtered to prevent overfitting, thus promoting generalization and robustness in the forecasting models. This method already shown promising results in avoiding overfitted models (31).

### 3.2 Piecewise polynomial regression

Piecewise Polynomial Regression (PRR) is the process of estimating a regression function by fitting multiple polynomial functions to different segments of the dataset. In this regression model, several polynomial functions are used to approximate the regression function in specific data segments (32).

PPR divides the dataset into segments and uses polynomial functions to approximate the regression function. The foundation of this segmentation is input space partitioning. Every segment is equivalent to a polynomial function that denotes the regression relationship inside that certain interval (32, 33).

To determine the optimal piecewise polynomial estimator, the research paper suggests considering various models defined by partitions and polynomial degrees. It utilizes a penalized least squares criterion to identify the model with an estimator closely resembling the best one in terms of quadratic risk. Additionally, the study establishes a non-asymptotic risk bound to ensure the selected model’s performance.

Extending the methodology to tree-structured partitions akin to those in the CART (34) algorithm offers a novel approach to constructing piecewise polynomial estimators for regression functions. This extension involves iteratively optimizing the selection of polynomial functions within each segment to best represent the underlying regression function across the entire data range.

### 3.3 Kernel ridge regression

The ideas of linear ridge regression are extended in the robust non-linear regression method known as kernel ridge regression (KRR). Utilizing the kernel trick, KRR adeptly captures the non-linear patterns inherent in data, rendering it applicable across diverse scenarios. The regularization mechanisms in KRR, including the ridge penalty, play a pivotal role in shaping the optimized kernel function, thereby mitigating overfitting concerns and bolstering predictive precision (35, 36).

Assume we have a dataset ${\left\{\left(x_{i},y_{i}\right)\right\}}_{i=1}^{N}$ consisting of *N* data rows that have been sampled from a distribution ℙ over the Cartesian product of *X* and the real numbers (ℝ). The objective is to identify the best function that, with the expectation computed collectively over *(X,Y)* pairs, minimizes the Mean Squared Error (MSE) of the $\mathrm{data}{\left(f\left(x\right)-y\right)}^{2}$ .

The conditional mean $f^{*}\left(x\right):=E\left[Y|X=x\right]$ is widely regarded as the most suitable function (37). Using a squared Hilbert norm penalty and the M-estimator with the lowest squares loss on the dataset is a feasible approach to forecast the unknown function $f^{*}$ (37).

$$\hat{f}:=\underset{f\in H}{\mathrm{argmin}}\left\{\frac{1}{N}\overset{N}{\underset{i=1}{\sum }}{\left(f\left(x_{i}\right)-y_{i}\right)}^{2}+⋋{\left|\left|f\right|\right|}_{H}^{2}\right\}\text{,}$$

Here, H denotes a reproducing kernel Hilbert space and ⋋ > 0 represents a regularization parameter. The kernel ridge regression estimate (referred to as KRR) serves as the estimator employed in the equation above (38).

### 3.4 Tweedie regression

The Tweedie regression model is a versatile tool for analyzing data that is non-negative, right-skewed, and has a high probability of being zero (39). It provides a single model that can effectively handle various types of continuous data automatically, making model selection easier during fitting.

The Tweedie regression model is predicated upon the assumption that the response variable *Y* follows a Tweedie distribution, denoted as $Y∼Tw_{p}\left(u,\phi ,p\right)$ , where *u* represents the mean, ϕ denotes the dispersion parameter, and *p* signifies the power parameter. This model postulates a relationship between the mean and variance of *Y*, where the variance is proportional to the mean raised to the power parameter *p*, expressed as $Var\left(Y\right)=\phi \cdot u^{p}$ . The regression parameters b are linked to the mean of *Y* through a specified link function $g\left(\cdot \right)$ , such that $E\left(Y\right)=g\left(Xb\right)$ , where *X* represents the design matrix. The likelihood function for the Tweedie regression model involves the probability density function of the Tweedie distribution, which entails complexity due to the presence of the power parameter *p*, necessitating numerical methods for its evaluation. Estimation of the regression parameters *b* and the dispersion parameter ϕ typically relies on maximum likelihood, quasi-likelihood, or pseudo-likelihood methods, leveraging second-moment assumptions to facilitate efficient and adaptable modeling of continuous data (39, 40).

## 4 Results and discussion

In this study, we implemented regression models to predict the solubility of ketoprofen in supercritical CO₂ using the provided dataset. The dataset includes measurements of temperature (T in Kelvin) and pressure (P in bar) as inputs, and solubility as the output. The regression models employed are PPR, KRR, and TDR. To optimize the hyperparameters of these models, we utilized the Water Cycle Algorithm (WCA), a robust optimization method known for its efficiency in finding optimal solutions in complex search spaces. The dataset was partitioned into training and testing subsets (80% training and 20% test) randomly, and the effectiveness of the models was gaged using a diverse set of metrics. The models are implemented on a machine with core-i7 CPU and 8Gb RAM which takes very small time (near Realtime) for each model to be executed. The effectiveness of regression models was appraised based on the following criteria (41):

1. *R*^2^ Score (Coefficient of Determination):
$$R^{2}=1-\frac{\sum_{i=1}^{n}{\left(Y_{\mathrm{observed},i}-Y_{\mathrm{predicted},i}\right)}^{2}}{\sum_{i=1}^{n}{\left(Y_{\mathrm{observed},i}-\bar{Y_{\mathrm{observed}}}\right)}^{2}}$$

These metric measures how well model predictions match data. An R^2^ score near 1 indicates a highly accurate model.

1. Mean squared error
$$MSE=\frac{1}{n}\overset{n}{\underset{i=1}{\sum }}{\left(Y_{\mathrm{observed},i}-Y_{\mathrm{predicted},i}\right)}^{2}$$

The average squared difference between the outcomes as seen in reality and those the model predicts is known as MSE. Lower MSE values indicate better model performance.

1. Mean absolute error
$$MAE=\frac{1}{n}\overset{n}{\underset{i=1}{\sum }}\left|Y_{\mathrm{observed},i}-Y_{\mathrm{predicted},i}\right|$$

Without taking into account the direction of the errors, MAE determines the average magnitude of errors in a set of predictions. Like MSE, lower MAE values indicate better model performance.

**Figure 4:**
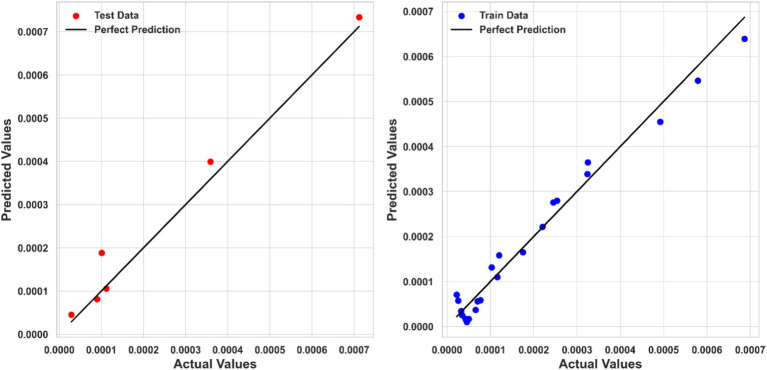
Actual and predicted values comparison (PPR model).

Table 2 summarizes the numerical findings for every regression model. The results indicate that Piecewise Polynomial Regression (PPR) outperforms both Kernel Ridge Regression (KRR) and Tweedie Regression (TDR) across all evaluation metrics. PPR achieved the highest R^2^ score of 0.97111, indicating that it explains approximately 97% of the variance in solubility. Additionally, PPR had the lowest MSE (1.6867E-09) and MAE (3.01040E-05), further demonstrating its superior predictive accuracy. Figure 4 shows a comparison of predicted solubility values and their corresponding actual values using the PPR model.

**Table 2:** Performance metrics for the regression models predicting ketoprofen solubility.

Model	R^2^ Score	MSE	MAE
PPR	0.97111	1.6867E-09	3.01040E-05
KRR	0.95044	2.5499E-09	3.49707E-05
TDR	0.84413	7.4249E-09	5.69159E-05

The results obtained in this study showed superior performance compared to the previous machine learning models developed for prediction of drug solubility in supercritical CO_2_. The accuracy reported for machine learning modeling of Hyoscine solubility in supercritical CO_2_ was reported to be 2.1680E-01 for the best model in terms of RMSE which is higher than our model developed in this study (20). A RMSE value of 2.74912E-01 was reported by Almehizia et al. (42) for prediction of multiple drugs solubility in supercritical CO_2_. The best model was reported to be HS-PR (Harmony Search-Polynomial Regression).

**Figure 5:**
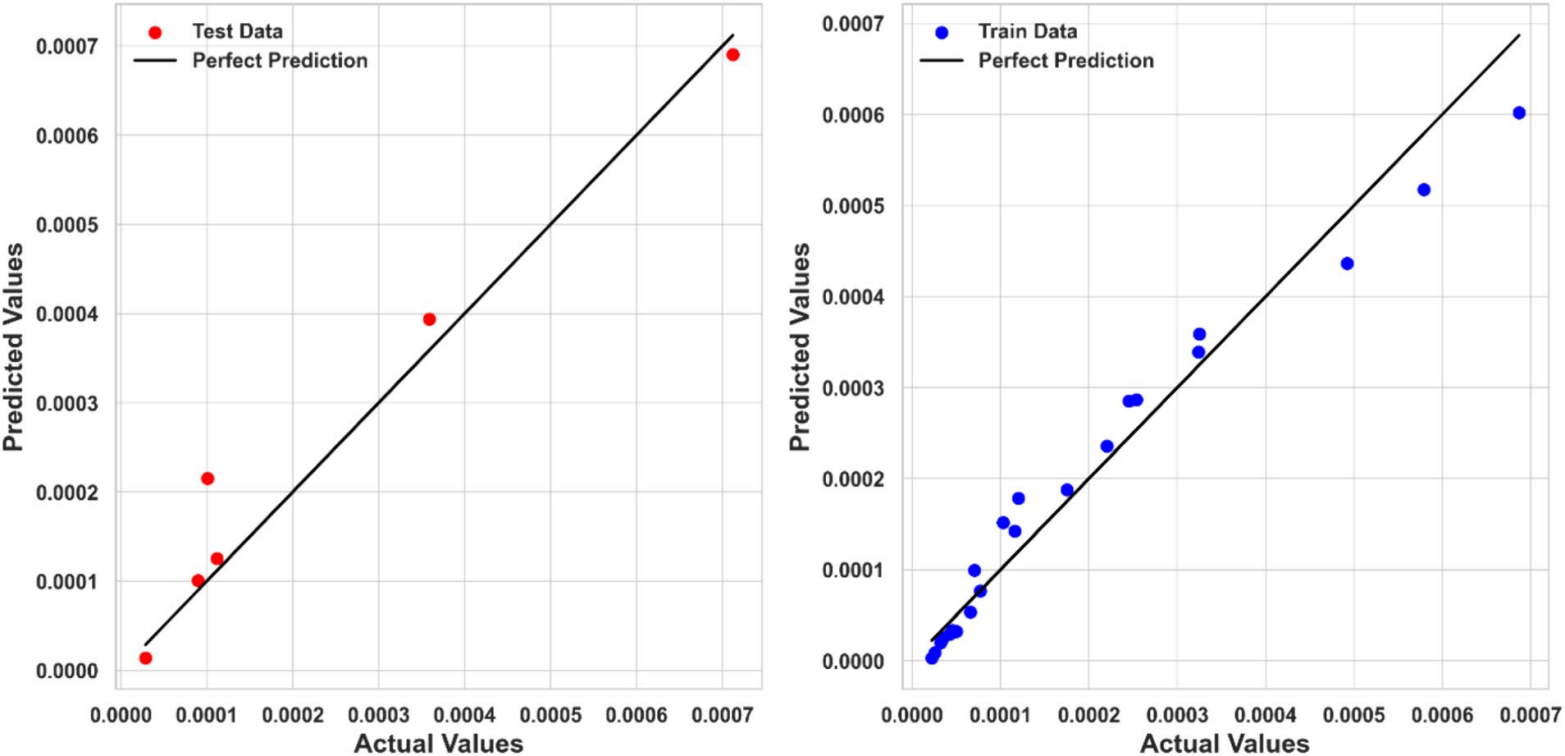
Actual and predicted values comparison (KRR model).

Kernel Ridge Regression (KRR) also performed well, with an R^2^ score of 0.95044, an MSE of 2.5499E-09, and an MAE of 3.49707E-05. Although KRR’s performance was slightly inferior to PPR, it still showed strong predictive capabilities, making it a viable alternative for modeling ketoprofen solubility. Figure 5 compares the KRR model-predicted and corresponding actual solubility values.

**Figure 6:**
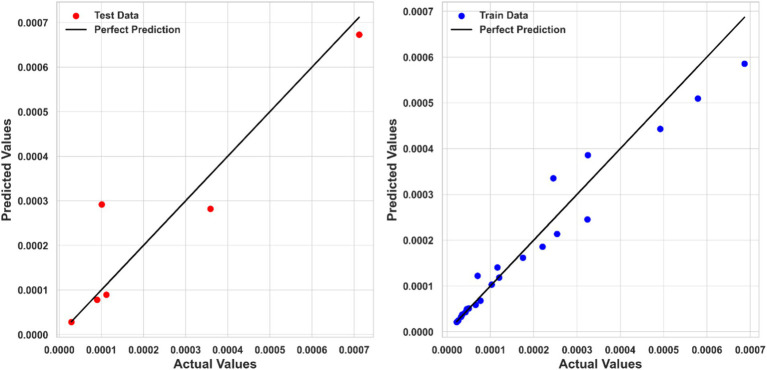
Actual and predicted values comparison (TDR model).

Tweedie Regression (TDR) exhibited the lowest performance among the three models, with R^2^ score of 0.84413, an MSE of 7.4249E-09, and an MAE of 5.69159E-05. Despite its relatively lower performance, TDR provided reasonable predictions, but it is clear that more complex models like PPR and KRR better capture the relationship between input features and solubility. The comparison between predicted and actual solubility values using the TDR model is depicted in Figure 6. Although some deviations can be observed in the testing step for this model, the overall fitting accuracy is acceptable considering the complexity of the process and dataset. Moreover, the models have been optimized in a way to minimize the risk of overprediction for the test dataset.

**Figure 7:**
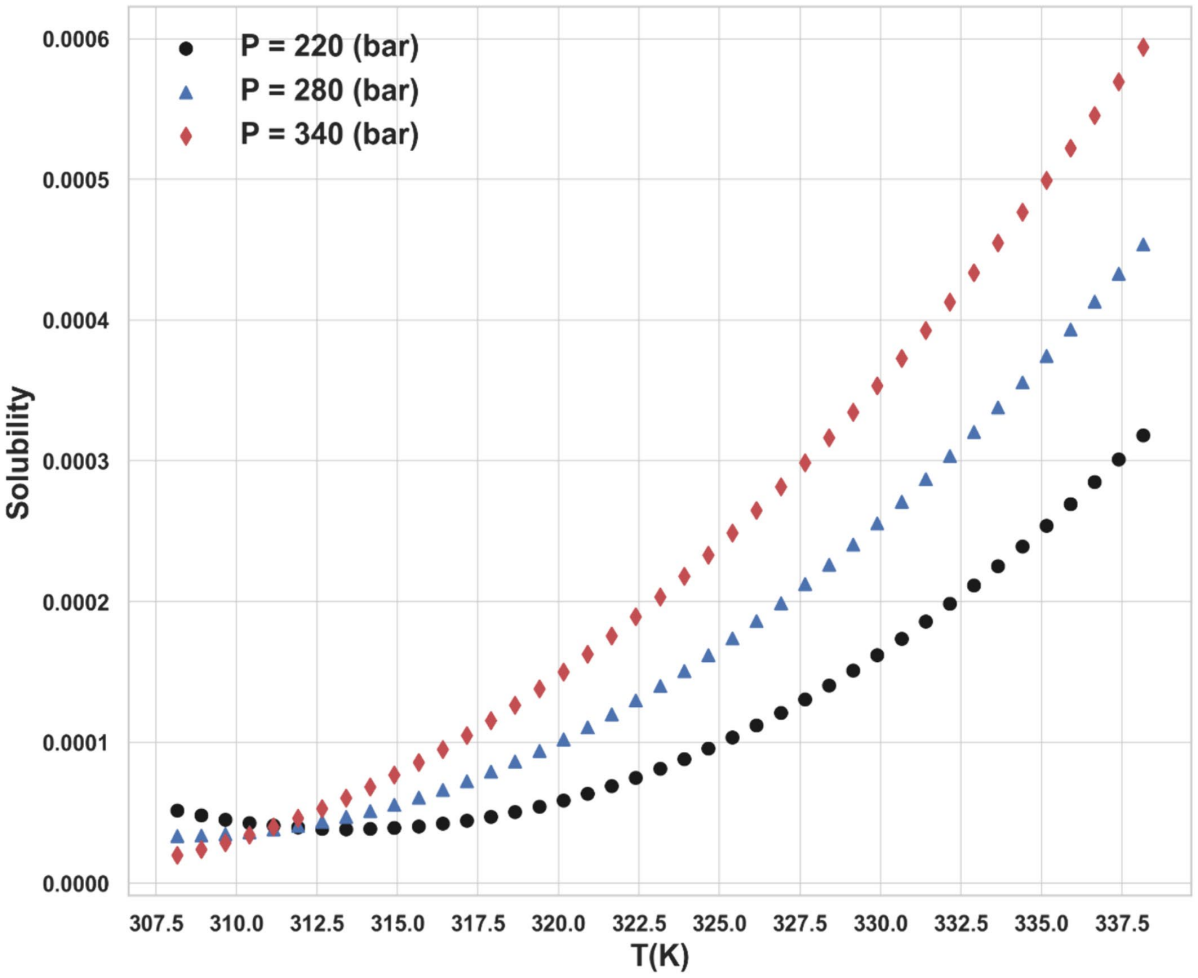
Impact of temperature on the solubility on different pressure levels.

**Figure 8:**
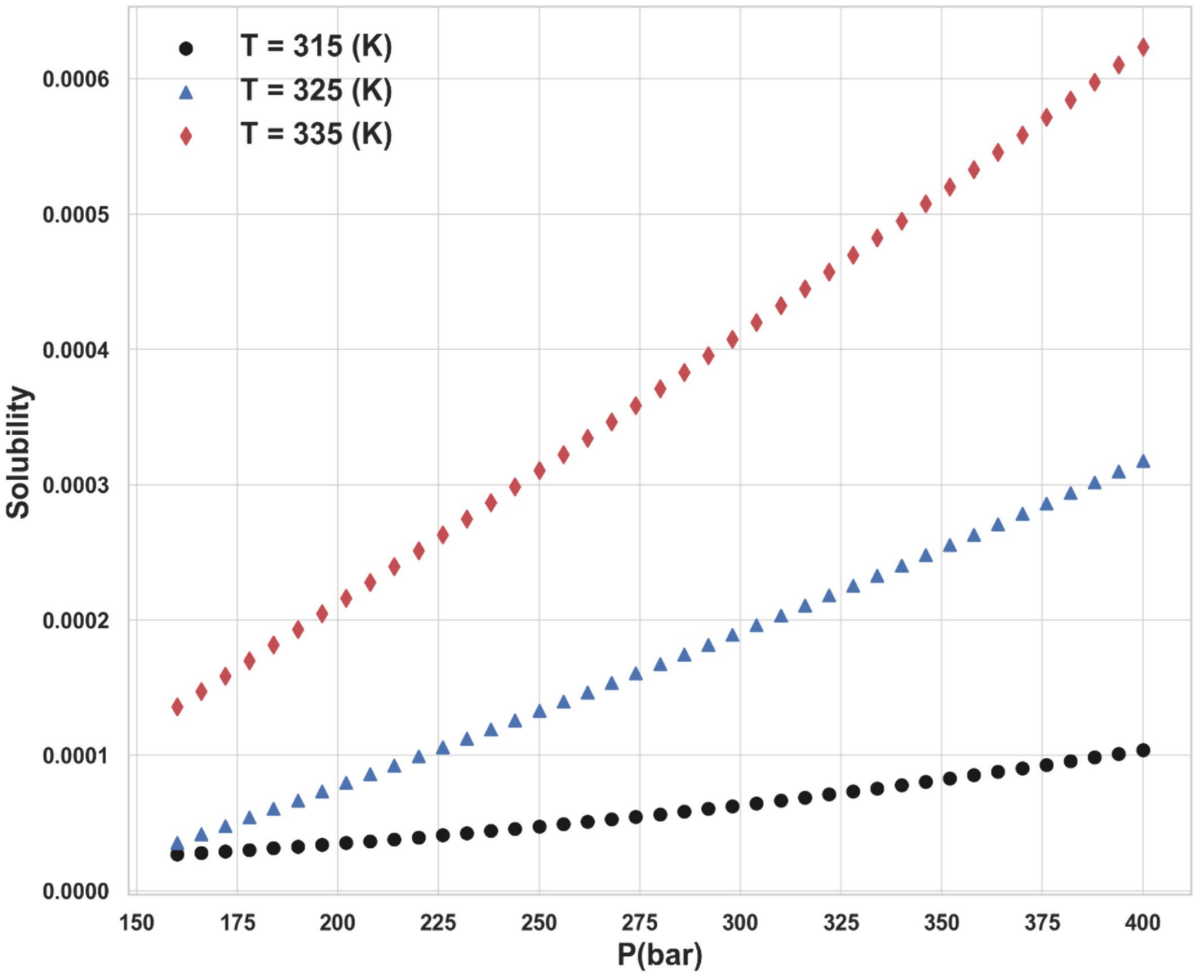
Impact of pressure on the solubility on different temperature levels.

**Figure 9:**
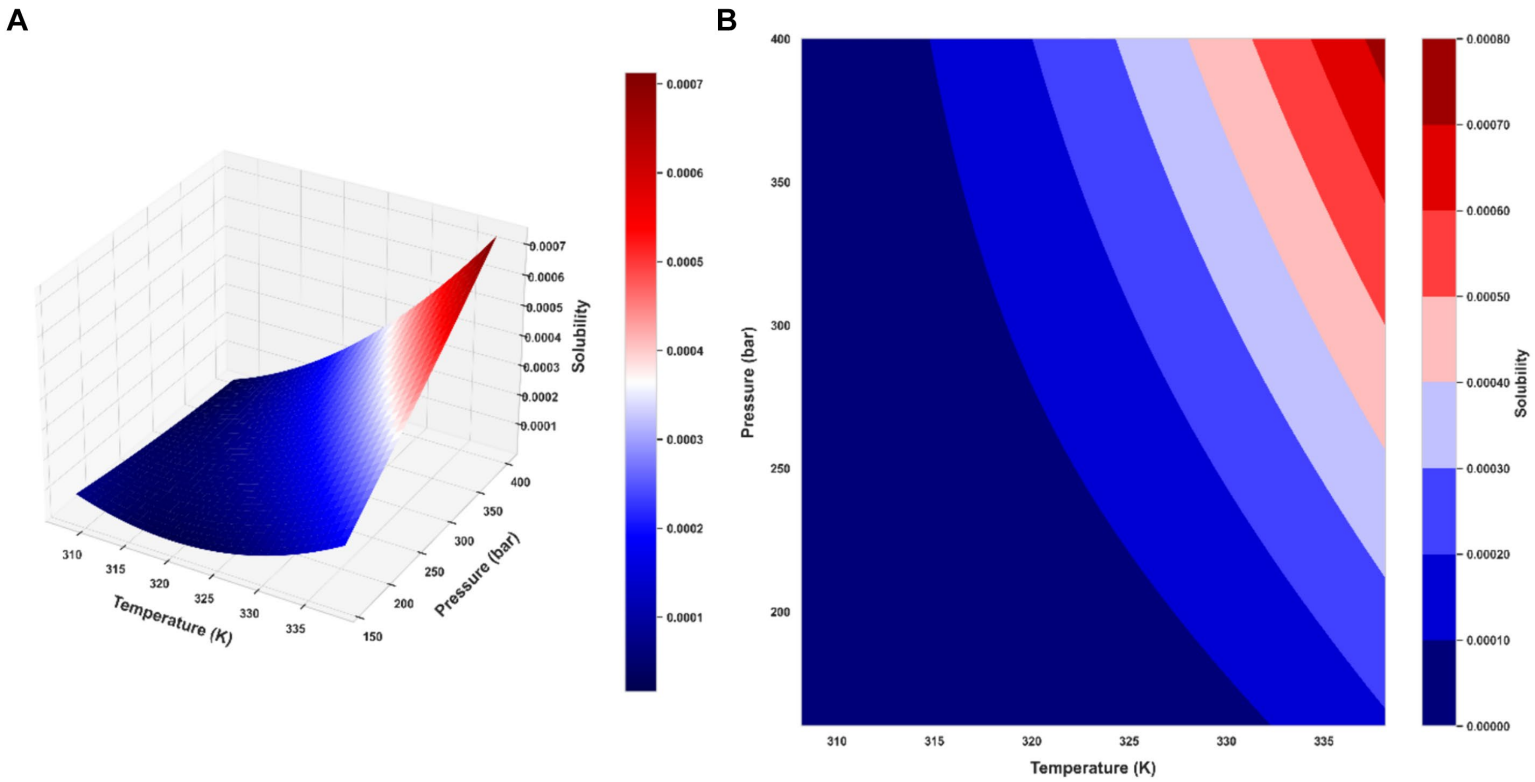
Final PPR model: **(A)** the 3D representation of predicted solubility values **(B)** contour plot of predicted solubility values.

Ultimately, the Piecewise Polynomial Regression (PPR) model shows excellent accuracy and dependability in predicting the solubility of ketoprofen in supercritical CO₂. Comparing this model to the other models assessed in this work, it performs better because it can partition the data space and fit polynomial functions within each segment to efficiently capture the underlying patterns in the data. Figures 7, 8 illustrate, with this model, how inputs affect the solubility values. Furthermore, shown in Figure 9 is the solubility in a contour plot and three-dimensional manner as a function of T(K) and P(bar). The trend for temperature shows exponential increase of solubility with temperature rise. On the other hand, a linear trend was observed for the influence of pressure on the drug solubility (see Figure 8). Thus, the maximum amount of ketoprofen solubility is determined at the highest values of T and P based on the ML models. Indeed, there is no optimum point for the solubility, and the optimum conditions should be determined from the process cost and economic evaluations.

## 5 Conclusion

In this study, we successfully developed and evaluated three machine learning regression models–PPR, KRR, and TDR–to predict the solubility of ketoprofen in supercritical CO_2_. By employing the Water Cycle Algorithm for hyperparameter tuning, we optimized each model’s performance, demonstrating the effectiveness of this approach in enhancing predictive accuracy. Our comparative analysis revealed that PPR outperformed the other models, providing the most accurate predictions with an *R*^2^ score of 0.97111, a MSE of 1.6867 × 10^−9^, and a MAE of 3.01040 × 10^−5^. This paper makes multiple contributions. Initially, we assess the three regression models used for predicting ketoprofen solubility, emphasizing their advantages and disadvantages. Furthermore, we illustrate the enhancement of model performance through the optimization of hyperparameters using the Water Cycle Algorithm. Ultimately, we provide comprehensive visual representations and statistical examinations to uncover the connections between temperature (T), pressure (P), and solubility. This study employs sophisticated machine learning techniques and resilient optimization strategies to forecast the solubility of supercritical fluids and enhance the optimization of pharmaceutical processes.

## Data availability statement

The original contributions presented in the study are included in the article/supplementary material, further inquiries can be directed to the corresponding author/s.

## Author contributions

TH: Conceptualization, Validation, Formal analysis, Writing – Review & Editing. SA: Supervision, Validation, Investigation, Resources, Writing – Review & Editing. SB: Conceptualization, Formal analysis, Investigation, Methodology, Resources, Validation, Visualization, Writing – original draft, Writing – review & editing.
